# Network pharmacology and molecular docking: combined computational approaches to explore the antihypertensive potential of Fabaceae species

**DOI:** 10.1186/s40643-024-00764-6

**Published:** 2024-05-20

**Authors:** Zainab Shahzadi, Zubaida Yousaf, Irfan Anjum, Muhammad Bilal, Hamna Yasin, Arusa Aftab, Anthony Booker, Riaz Ullah, Ahmed Bari

**Affiliations:** 1 Department of Botany, Lahore College for Women University, Lahore, Pakistan; 2 Department of Basic Medical Sciences, Shifa College of Pharmaceutical Sciences, Shifa Tameer-e-Millat University, Islamabad, Pakistan; 3 Centers for Applied Molecular Biology, University of the Punjab, Lahore, Pakistan; 4 Research Centre for Optimal Health, School of Life Sciences, College of Liberal Arts and Sciences, University of Westminster, 115 New Cavendish Street, London, W1W 6UW UK; 5 Research Group ‘Pharmacognosy and Phytotherapy’, UCL School of Pharmacy, Univ. London, 29 - 39 Brunswick Sq., London, WC1N 1AX UK; 6 Department of Pharmacognosy, College of Pharmacy King, Saud University, Riyadh, Saudi Arabia; 7 Department of Pharmaceutical Chemistry, College of Pharmacy King, Saud University, Riyadh, Saudi Arabia

**Keywords:** Hypertension, Herbal medicine, Phytochemicals, Fabaceae, *Cassia* species, Network pharmacology, Molecular docking

## Abstract

Hypertension is a major global public health issue, affecting quarter of adults worldwide. Numerous synthetic drugs are available for treating hypertension; however, they often come with a higher risk of side effects and long-term therapy. Modern formulations with active phytoconstituents are gaining popularity, addressing some of these issues. This study aims to discover novel antihypertensive compounds in *Cassia fistula*, *Senna alexandrina*, and *Cassia occidentalis* from family Fabaceae and understand their interaction mechanism with hypertension targeted genes, using network pharmacology and molecular docking. Total 414 compounds were identified; initial screening was conducted based on their pharmacokinetic and ADMET properties, with a particular emphasis on adherence to Lipinski's rules. 6 compounds, namely Germichrysone, Benzeneacetic acid, Flavan-3-ol, 5,7,3',4'-Tetrahydroxy-6, 8-dimethoxyflavon, Dihydrokaempferol, and Epiafzelechin, were identified as effective agents. Most of the compounds found non-toxic against various indicators with greater bioactivity score. 161 common targets were obtained against these compounds and hypertension followed by compound-target network construction and protein–protein interaction, which showed their role in diverse biological system. Top hub genes identified were TLR4, MMP9, MAPK14, AKT1, VEGFA and HSP90AA1 with their respective associates. Higher binding affinities was found with three compounds Dihydrokaempferol, Flavan-3-ol and Germichrysone, −7.1, −9.0 and −8.0 kcal/mol, respectively. The MD simulation results validate the structural flexibility of two complexes Flavan-MMP9 and Germich-TLR4 based on no. of hydrogen bonds, root mean square deviations and interaction energies. This study concluded that *C. fistula* (Dihydrokaempferol, Flavan-3-ol) and *C. occidentalis* (Germichrysone) have potential therapeutic active constituents to treat hypertension and in future novel drug formulation.

**Graphical Abstract:**

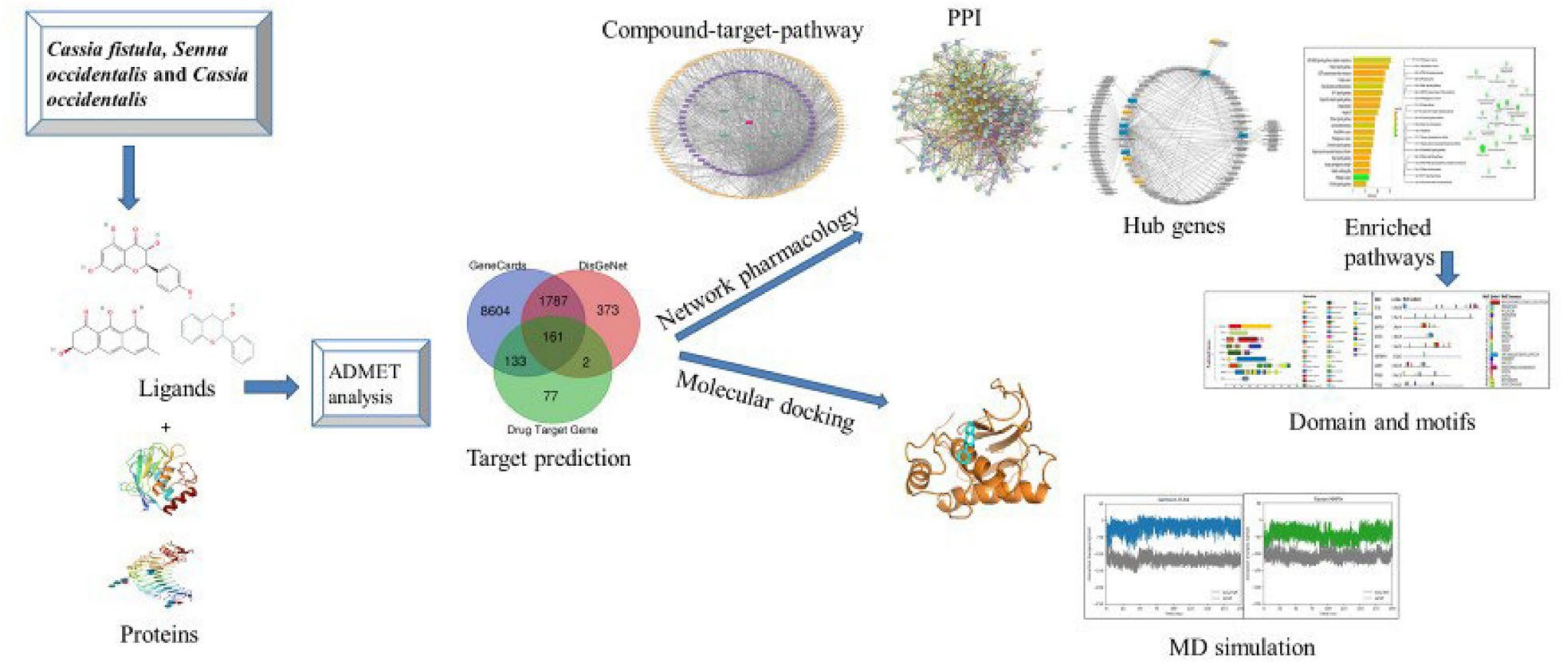

## Background

Hypertension is commonly known as high blood pressure. It is a globally prevalent and highly detrimental chronic medical condition characterized by consistently elevated blood pressure within arteries. It significantly pose high health risks (Oparil et al. 2018; Liao et al. 2023). It is a leading factor for various cardiovascular diseases, including cardiac arrest, coronary artery disease, strokes, ischemia, vision loss, and renal diseases (Wang et al. 2021; Yang et al. 2023). This hypertension related consequences are responsible for 9.4 million deaths worldwide. It is estimated that this count will exceed to 1.56 billion by the year 2025 (Zhai et al. 2021). Hypertension often develops silently, without noticeable symptoms. Hence, regular blood pressure monitoring is crucial for early detection and management (Hasanzadeh et al. 2023). According to two empirical studies in Pakistan, which are based on National Health Survey and rural northern areas of the country, hypertension prevalence rate is 46.2% and 33%, respectively (Almas et al. 2023; Shah et al. 2023). If this number continues to increase then one out of every third person will be a victim of hypertension (Elahi et al. 2023). Synthetic medication such as diuretics, angiotensin receptor blockers, angiotensin-converting enzyme inhibitors, anti-adrenergic drugs, and calcium channel blockers have demonstrated positive outcomes in the management of hypertension in patients but these drugs have side effects as well (Mancia et al. 2019; Zhan et al. 2023). Therefore, this sort of medications requires double therapy. So it doubled the cost of medication, therefore, it is essential to develop monotherapy options with less side effects (Karr 2017; Juwita et al. 2023).

Moreover, exploring the potential of natural products through a reverse pharmacology approach while prioritizing safety profiles may represent a rational strategy to treat hypertension. In this context, medicinal plants continue to hold immense importance for humanity due to their contribution to the development of modern medicines in the healthcare sector (Jasemi et al. 2020; Qamar et al. 2023). The family Fabaceae is one of the largest family of angiosperm. Several species of this family have traditionally been used to treat hypertension (Asfaw and Abebe 2021). *Cassia fistula*, *Senna alexandrina* and *Cassia occidentalis* are the three tropical and subtropical trees belongs to this family and are native to eastern Australia, southern Africa, Hawaii, southern South America, Indian subcontinent, South East Asia, Saudi Arabia, Egypt and Yemen (Sharma et al. 2021; Natarajan et al. 2022) These plants are abundant in secondary metabolites, including tannins, phenolics, alkaloids, terpenoids, flavonoids and cardiac glycosides (Shailajan et al. 2013; Naz et al. 2020). They exhibit a diverse range of pharmacological properties, including analgesic, cardioprotective, anti-inflammatory, antioxidant, antidiabetic, hypoglycemic, and hepatoprotective activities (Thomford et al. 2018; Murugesan et al. 2019). Plant extract treatments encounter safety and dosage challenges. Modern formulations involve active phytoconstituents are increasingly gaining popularity, effectively addressing these concerns (Persechino et al. 2022).

Insilico drug discovery methods, which predict compound efficacy against a range of diseases, hold promising techniques that can accelerate drug development and decrease costs by reducing the necessity for extensive laboratory experiments (Gupta et al. 2023). Network pharmacology approach has gained prominence over the years, offering a holistic approach to constructing 'protein-compound/disease-gene' networks for identifying concurrent treatment pathways (Zhou et al. 2020; Xin et al. 2021). These methods are also valuable for predicting compound toxicity, drug classification, and bioactivity. Researchers often combine network pharmacology with molecular docking to understand drug-target interactions, predict potential drug candidates more effectively and accelerate the drug discovery process (Li 2021; Noor et al. 2022; Singh et al. 2022). Molecular docking is a crucial computational technique for predicting atomic-level interactions between small molecules and target proteins, aiding in rational drug design and the optimization of existing ones (Dey et al. 2023; Ibrahim et al. 2023).

The design of molecular docking programs has become essential in herbal drug discovery endeavors, particularly for conducting virtual screenings of phytochemicals or nutraceuticals to identify potential therapeutic compounds (Agu et al. 2023). Herbal drugs face efficacy and standardization problems. So far, in literature this sort of computational studies related to the Fabaceae species particularly, *Cassia fistula*, *Senna alexandrina*, and *Cassia occidentalis* has not been discovered. Hence, there is need of advanced exploration based on computational techniques to evaluate natural Fabaceae compounds for hypertension treatment, combining network pharmacology and molecular docking to identify lead compounds and their mechanisms of action. This will explore potential of Fabaceae-derived natural products as alternative antihypertensive agents, expedite the drug discovery. This study also aims to understand their potential mechanisms for treating hypertension as well as binding affinities between ligands and protein complexes.

## Materials and methods

### Screening of active compounds

The phytoconstituents of three plant species, namely *Cassia fistula*, *Senna alexandrina*, and *Cassia occidentalis*, were extracted by a thorough review of published literature and different databases. Various databases, such as Google Scholars, PubChem (https://pubchem.ncbi.nlm.nih.gov/ accessed 25 September 2023), IMPPAT (https://cb.imsc.res.in/imppat/ accessed on 25 September 2023), and Phytohub (https://phytohub.eu/ accessed on 25 September 2023), were employed for this purpose. The 3D structures and physiochemical characteristics of the identified compounds were sought through resources like PubChem (https://pubchem.ncbi.nlm.nih.gov/ accessed on 27 September 2023), SpiderChem (http://www.chemspider.com/ accessed on 27 September) and NIST Library (https://webbook.nist.gov/chemistry/# accessed on 27 September 2023). This was achieved by referencing compound names, formulas, and CID/SID numbers. Subsequently, the Canonical SMILES notation was utilized to explore the pharmacokinetic properties of all active compounds (Sarkar et al. 2023).

### Compound/ligand selection through pharmacokinetic properties and ADMET analysis

The compound/ ligand pharmacokinetic properties were finding out by using software DataWarrior V5.5.0 (accessed on 28 September 2023). Lipinski’s rule of five for drug discovery was considered the standard criteria for pharmacokinetic properties i.e. compounds that encompasses oral bioavailability (OB ≥ 30), molecular weight (MW < 500 Da), drug Likeness (DL ≥ 0.18), hydrogen bond donors (H donor < 5), octanl water coefficient (P < 5) and hydrogen bond acceptors (H acceptor < 10) are ideal for study (Daina et al. 2017; Shahid et al. 2022). The ADMET (absorption, distribution, metabolism, excretion, and toxicity) properties of all compounds were predicted using two online software, SwissADME (http://www.swisstargetprediction.ch/ accessed on 28 September 2023) and ADMETlab 2 (https://admetmesh.scbdd.com/ accessed on 28 September 2023). These programs assess key pharmacokinetic characteristics of a compound/ligand, including its interaction with the blood–brain barrier (BBB), distribution, absorption in the gastrointestinal tract, metabolism as a substrate for P-glycoprotein (P-gp), inhibition of cytochrome P450 enzymes such as CYP1A2, CYP2C19, CYP2C9, CYP2D6, CYP3A4, and lipophilicity for absorption through the plasma membrane (Mukhtar and Khan 2023).

### Compounds toxicity assessment

Drug toxicity refers to the harmful effects of a substance when taken in excessive amounts or when the body is unable to metabolize and eliminate it properly. It can range from mild side effects to severe, life-threatening reactions. Two software was utilized, DataWarrior V5.5.0 (accessed on 29 September 2023) and Protox II server (https://tox-new.charite.de/protox_II/ accessed on 29 September 2023), for the prediction of various toxicity indicators including carcinogenicity, Immunotoxicity, Irritating effect, reproductive, hepatotoxicity, and mutagenicity. The understudy compounds were also subjected to assessment for predicting their LD_50_ values and drug toxicity classifications. LD_50_ values are commonly expressed in mg/kg of body weight and represent the dose at which 50% of test subjects succumb after exposure to a substance. Toxicity classes are defined in accordance with the Global Harmonization System (GHS) for the categorization and labeling of substances (Nafisah et al. 2022).

### Bioactivity score prediction

The drug score values serve as an indicator of the inherent potential of a prospective complex to function as a potential drug candidate. Using the web-based tool Molinspiration (https://www.molinspiration.com/ accessed on 1st October 2023), predictions were made regarding the bioactivity score of phytoconstituents concerning their interaction with human receptors, including G protein-coupled receptors (GPCRs), kinases, proteases, ion channels, enzymes, and nuclear receptors. A compound is classified as dynamic (active) if its bioactivity score exceeds 0.0, moderately active if it falls within the range of -5.0 to 0.0, and inactive if the bioactivity score is below −5.0 (Mukhtar and Khan 2023).

### Network pharmacology profiling of compounds

#### Potential target screening of active compounds and hypertension

The data of potential targets for active compounds were retrieved from SwissTargetPrediction (http://www.swisstargetprediction.ch/ accessed on 2nd October 2023) and STITCH (http://stitch.embl.de/ accessed on 2nd October 2023) through inputting the canonical SMILES and specifying species as “Homo sapiens”. Whereas, the hypertension targets were downloaded from GeneCard (http://www.genecards.org/ accessed on 2nd October 2023) and DesGenet (http://www.disgenet.org/ accessed on 2nd October 2023). The targets of these databases were merged and removed repetitions in targets. The common names of the targets were also searched from UniProtKB (https://www.uniprot.org/ accessed on 3rd October 2023). The mutual targets of compounds and hypertension were achieved through Venn diagram construction using Bioinformatics tool (https://bioinformatics.psb.ugent.be/webtools/Venn accessed on 4 October 2023) (Tabassum et al. 2022).

#### Construction of compound-target network

The compound-targets network was constructed to check the interaction of active compounds within the complex biological system by using Cytoscape V3.10.1 (https://cytoscape.org/ accessed on 4 October 2023). In this network, nodes symbolize the chemical constituents and targets, with edges illustrating their interactions. The network analyzer function was utilized to evaluate the fundamental characteristics of the network. Following this, the network underwent filtering based on the "degree," which represents the number of connected nodes linked to a specific network node as a node attribute (Ram et al. 2023).

#### Prediction of protein–protein-interaction network and hub genes

The protein–protein Interaction of 161 common genes was assessed through STRING database (https://string-db.org/ accessed on 5 October 2023), with the organism specified as "Homo sapiens. The protein–protein Interaction network was visualized using Cytoscape V3.10.1 (accessed on 5 October 2023). CytoHubba plugin was used to identify the hub genes and nodes exhibiting elevated degrees within the network. The strong associations of the genes being targeted are emphasized by the prominence of the highest degree (Tao et al. 2013).

#### Construction of target–compound–pathway network

The data for KEGG pathway analysis of the top hub genes was obtained from the DAVID database (https://david.ncifcrf.gov/tools.jsp accessed on 6 October 2023) and network was constructed to check the compounds mechanism in these pathways (Tabassum et al. 2022).

#### Gene ontology and KEEG pathway analysis

The Gene Ontology and KEEG pathway analysis was performed by using functional genes annotation resource database DAVID (http://david.ncifcrf.gov/ accessed on 7 October), with specified organism “Homo sapiens”. It employs Gene Ontology analysis to classify gene functions into biological processes (BP), cellular components (CC), molecular functions (MF) and enrichment pathway analysis into KEEG pathway. The cut off method with a probability score below 5 × 10^–2^ was applied to select the top 20 GO annotations (BP, CC and MF) and KEEG pathways to draw bar and lollipop plot by using Shiny GO (http://bioinformatics.sdstate.edu/go/ accessed on 6 October 2023).

#### Domain and motif analysis

Domain and motif analysis were performed by using two databases: NCBI-CDD (https://www.ncbi.nlm.nih.gov/Structure/cdd/cdd.shtml accessed on 9 October 2023) and MEME (https://meme-suite.org/meme/db/motifs accessed on 10 October 2023), respectively.

### Molecular docking

Molecular docking simplifies the investigation of interactions between ligands and proteins, making it possible to discover their respective associates. Active components' 3D structures were extracted from PubChem in SDF format and optimized, while potential genes' structures were obtained from RCSB PDB in PDB format (https://www.rcsb.org/ accessed on 7 October 2023) while selecting the best protein crystal structure for docking, emphasizing smaller resolution, completeness, and human origin. Protein structure was refined by using software PyMOL V2.5.5 to remove ligands and water molecules. Following this, the ligand and protein molecules were subject to a series of operations, including charging, hydrogenation, and normalization, using AutoDockTool V1.5.6, culminating in the generation of PDBQT file formats (Mir et al. 2023a, b). The interaction between the processed ligands and proteins was subsequently examined through molecular docking with AutoDock Vina. AutoDock Vina, renowned for its user-friendliness, rapid processing speed, automated grid box dimension calculation, and convenient estimation of binding sites. AutoDock Vina facilitated the incorporation of phytoligands, which were treated as "flexible," into protein targets that were considered "rigid” (Mukhtar and Khan 2023). In this study, a grid box size of 38 × 44 × 56 (x, y, and z) with a grid spacing of 0.375 was employed for Flavon-3-ol. The grid center was positioned at coordinates 1.417, 47.278, and 21.667 for x, y and z. For Dihydrokaempferol, the grid box was created with size 36 × 38 × 44 xyz points, grid spacing of 0.375 Å and grid center of x, y and z dimensions of 19.500, −11.806 and 10.083, respectively. For Germichrysone, the grid box was set at 66 × 32 × 28 xyz points with grid spacing of 0.375 Å and grid center was designated at dimensions (x, y and z): 5.750, −0.500 and 0.333, respectively. To calculate the binding energy associated with these interactions, Command Prompt on Microsoft Window V6.3.900 was utilized, facilitating the visualization of the docking results. The docking search parameters employed include Lamarckian Genetic Algorithm, with the number of genetic algorithm runs ranging from 10 to 100 in increments of 10. The population size is set at 150, while the maximum number of energy evaluations is moderate at 2,500,000. Additionally, the maximum number of generations is set to 27,000, with default docking parameters utilized for run. Scoring functions were employed to evaluate and rank the poses produced throughout the docking procedure. These functions gauge the binding free energy or affinity between the ligand and receptor (Durhan et al. 2022).

#### Molecular dynamic simulation

Molecular dynamic (MD) simulation was performed to find out the stability and variability of top ranked docking complexes. Top scoring protein–ligand complexes were simulated to determine the binding affinities of the best hit compounds after docking by using software GROMACS version 2020 (Release 2017) with specific system (Lenovo ThinkSystem SR650; Processor: 2 × Intel(R) Xeon (R) Gold 6130 CPU @ 2.10 GHz (32 Cores); RAM: 4 × 32 GB DDR4; Drivers: 1 × 1 TB NVMe; 2 × 4 TB SAS RAID). The protein topology and parameters for MD simulation was generated using the charm 3 force field and CGenFF server (Mazurek et al. 2021; Ko et al. 2022). The TIP3P water model was used for solvating each system, followed by neutralization with the requisite quantities of Na^+^ and Cl^−^. Then, the energy of each system was minimized using the steepest descent minimization algorithm with a maximum of 50,000 iterations and < 10.0 kJ/mol of force. Position constrains were applied to the receptor and ligand of both systems for 100 ps during leapfrog integrator, a 2 fs time step, and LINCS holonomic constrains. The NPT (Number of Atoms, Pressure, and Temperature) ensembles were used for 100 ps at temperature (300 K) with a 2 fs time step during the NPT equilibration phase. Following the energy minimization and equilibration of all the systems, an MD production run of 50 ns with a time step of 2 fs was performed, and the structure’s coordinates were saved every 10 ps. The trajectories were used for different dynamics evaluations after a 50 ns MD simulation, including root mean square deviation (RMSD) of ligands relative to the backbone of proteins. The amount of H-bonds between the ligand and proteins was estimated over a 50-ns period. The Coul-SR and LJ-SR ligand–protein interaction energies were also calculated.

#### Determination of binding free energies of the protein and ligand complexes by MM-PBSA

The protein–ligand complexes binding free energies (ΔG_Bind_) were determined through the utilization of the molecular mechanics Poisson–Boltzmann surface area (MM-PBSA) method, employing the adaptive Poisson–Boltzmann solver 3.0 (APBS 3.0) within the g_mmpbsa package (Gogoi et al. 2021). Widely acknowledged as one of the most employed techniques for calculating interaction energies within biomolecular complexes, the MM-PBSA approach, coupled with molecular dynamics (MD) simulation, allows for the elucidation of significant conformational fluctuations and entropic contributions to the binding energy. In essence, the binding free energy (G_Bind_) between a protein and a ligand in a solvent can be defined as follows:

$$\Delta {\text{G}}_{{{\text{Bind}}}} \, = \,\Delta {\text{H}}\, - \,{\text{T}}\Delta {\text{S}}$$

where ΔG_Bind_ represents the changes in Gibbs free energy, ΔH represents the change in enthalpy (heat), T is temperature in Kelvin and ΔS change in entropy (Kupferschmidt and Cohen 2020).

## Results and discussion

### Screening of active compounds

The utilization of computational screening and prediction to identify phytoconstituents possessing favorable pharmacodynamic and pharmacokinetic characteristics offers a time-efficient and cost-effective approach (Siddiqui et al. 2022). In this study, A total of 414 compounds were found reported in literature across three species *C. fistula*, *Senna alexandrina* and *C. occidentalis*. The compounds were initially screened through the examination of their pharmacokinetic properties and ADMET analysis. Six compounds including germichrysone, benzeneacetic acid, Flavan-3-ol, 5, 7, 3', 4'-Tetrahydroxy-6, 8-dimethoxyflavon, dihydrokaempferol and epiafzelechin, demonstrated effectiveness. Lipinski’s rule of five was also applied to conform the drug discovery criteria. According to this rule, all the 6 compounds have zero Lipinski’s rule violation and meet the standard criteria i.e. molecular weight (MW < 500 Da), Drug Likeness (DL ≥ 0.18), hydrogen bond donors (H donor < 5), octanl water coefficient (P < 5) and hydrogen bond acceptors (H acceptor < 10) (Table 1). An ideal drug is one that adheres to Lipinski’s rule without violations (Narkhede et al. 2020; Singh et al. 2023).

**Table 1:** Prediction of activity spectra for substances (PASS analysis) according to Lipinski’s rule of five

Sr. No	Compound name	3D structures	Molecular weight (g/mol) (MW < 500))	Drug likeness ((DL ≥ 0.18)	Oral bioavailability (OB ≥ 30)	clog P (p < 5)	H-bond acceptors (< 10)	H-bond donor (< 5)	No. of rotatable bonds (< 10)	Polar surface area (Å^2^) (≤ 140 Å^2^)	Lipinski’s rule of five violations
1	Germichrysone		258.27	0.71	0.55	2.26	4	3	0	77.76	0
2	Benzeneacetic acid		244.08	0.81	0.56	2.99	5	2	7	83.83	0
3	Flavan-3-ol		226.27	0.31	0.55	2.89	2	1	1	29.46	0
4	5,7,3',4'-Tetrahydroxy-6, 8-dimethoxyflavon		346.29	0.40	0.55	1.85	8	4	3	125.68	0
5	Dihydrokaempferol		288.25	0.44	0.55	1.30	6	4	1	107.22	0
6	Epiafzelechin		274.27	0.31	0.55	1.85	5	4	1	90.15	0

The investigation of ADMET properties for various compounds revealed that four substances, namely Germichrysone, 5,7,3',4'-Tetrahydroxy-6,8-dimethoxyflavon, Dihydrokaempferol, and Epiafzelechin, exhibited an incapacity to penetrate the blood–brain barrier. Conversely, Benzeneacetic acid and Flavan-3-ol demonstrated a high capability to traverse the blood–brain barrier. The blood–brain barrier is a protective barrier formed by endothelial cells in the blood vessels of the brain, which effectively blocks the entry of numerous toxins into brain tissues (Kadry et al. 2020; Alajangi et al. 2022). Two compounds Germichrysone and Epiafzelechin showed positive results for permeability glycoprotein substrates (P-gp substrates) while the remaining compounds showed negative efficacy. The results suggest that non-Pgp substrates exhibit improved persistence in their cells. The role of P-gp in drug transport is essential for pharmacology and drug development, as it can influence the bioavailability and efficacy of various medications (Karthika et al. 2022; Rachmale et al. 2022; Attia et al. 2023). In order to maintain consistent plasma concentrations and enhance the absorption of the tested compounds, it was expected that these substances would exhibit inhibitory actions on all five cytochrome P450 enzyme classes, namely CYP2C9, CYP2C19, CYP3A4, CYP1A2, and CYP2D6. Only one compound 5,7,3',4'-Tetrahydroxy-6,8-dimethoxyflavon showed inhibitory effect against CYP2C9, CYP3A4, CYP1A2, and CYP2D6. One compound flavan-3-ol showed inhibitory effect against CYP2D6. The remaining compounds exhibited no inhibitory activity against these cytochrome classes. Cytochrome P450 enzymes are a family of enzymes responsible for metabolizing a wide range of drugs and other xenobiotics (foreign substances) in the body. Inhibiting specific CYP enzymes can enhance drug bioavailability, extend half-life, and mitigate drug-drug interactions (Chatterjee et al. 2022; He et al. 2023; Xing et al. 2023). Each of the compounds demonstrated substantial gastrointestinal absorption, suggesting a pronounced capacity for absorption within the human intestinal tract (Table 2). Compounds with high gastrointestinal absorption exhibit efficient uptake and transport across the intestinal wall, enhancing their bioavailability (Azman et al. 2022).

**Table 2:** ADMET properties of Phytoconstituents

Phytoconstituents	CYP1A2 inhibitor	CYP3A4 Inhibitor	CYP2C19 inhibitor	CYP2D6 inhibitor	CYP2C9 inhibitor	BBB permeant	P-gp substrate	Log *K*_p_ (skin permeation)	GI absorption
Germichrysone	No	No	No	No	No	No	Yes	−7.46 cm/s	High
Benzeneacetic acid	No	No	No	No	No	Yes	No	−8.60 cm/s	High
Flavan-3-ol	No	No	No	Yes	No	Yes	No	−5.66 cm/s	High
5,7,3',4'-Tetrahydroxy-6, 8-dimethoxyflavon	Yes	Yes	No	Yes	Yes	No	No	−6.35 cm/s	High
Dihydrokaempferol	No	No	No	No	No	No	No	−7.13 cm/s	High
Epiafzelechin	No	No	No	No	No	No	Yes	−7.46	High

### Toxicity prediction of compounds

Toxicity assessment of compounds is a critical step in drug discovery, ensuring the safety and efficacy of potential therapeutic agents. Insilico tools for molecular docking offer a cost-effective and efficient means to predict drug toxicity, allowing researchers to evaluate potential drug candidates for their safety profiles before advancing to costly *Invitro* and *Invivo* experiments (Kumar et al. 2023; Sinha et al. 2023). Compound toxicity was assessed through a comprehensive analysis of six distinct toxicity factors, encompassing mutagenicity, reproductive toxicity, irritant potential, hepatotoxicity, carcinogenicity, Immunotoxicity, and cytotoxicity. All the compounds showed non-toxic effects against all the factors except two compounds (Germichrysone and 5,7,3',4'-Tetrahydroxy-6,8-dimethoxyflavon). Germichrysone showed highly toxic mutagenic and irritant effect and 5,7,3',4'-Tetrahydroxy-6,8-dimethoxyflavon showed highly toxic mutagenic and Immunotoxicity effect. With the exception of two compounds (Germichrysone and 5,7,3',4'-Tetrahydroxy-6,8-dimethoxyflavon), all the compounds exhibited non-toxic effects against various factors. Germichrysone displayed highly toxic mutagenic and irritant effects, while 5,7,3',4'-Tetrahydroxy-6,8-dimethoxyflavon exhibited highly toxic mutagenic and Immunotoxicity effects (Table 3).

**Table 3:** Toxicity prediction of effective compounds

Phytochemicals	Mutagenic	Reproduction	Irritant	Hepato-toxicity	Carcino-genic	Immuno-toxicity	Cyto-toxicity
Germichrysone	Highly toxic	Non-toxic	Highly toxic	Non-toxic	Non-toxic	Non-toxic	Non-toxic
Benzeneacetic acid	Non-toxic	Non-toxic	Non-toxic	Non-toxic	Non-toxic	Non-toxic	Non-toxic
Flavan–3–ol	Non-toxic	Non-toxic	Non-toxic	Non-toxic	Non-toxic	Non-toxic	Non-toxic
5,7,3',4'-Tetrahydroxy-6, 8-dimethoxyflavon	Highly toxic	Non-toxic	Non-toxic	Non-toxic	Non-toxic	Highly toxic	Non-toxic
Dihydrokaempferol	Non-toxic	Non-toxic	Non-toxic	Non-toxic	Non-toxic	Non-toxic	Non-toxic
Epiafzelechin	Non-toxic	Non-toxic	Non-toxic	Non-toxic	Non-toxic	Non-toxic	Non-toxic

### Prediction of LD_50_ and drug class

The level of toxicity varies according to the dosage, the short-term toxic impact is assessed through the median lethal dose (LD_50_) (Saganuwan 2017). Compounds LD_50_ and toxicity class prediction results showed that three compounds 5,7,3',4'-Tetrahydroxy-6,8-dimethoxyflavon, Flavan-3-ol and Epiafzelechin have drug toxicity class V (LD_50_ = 2500 mg/kg, LD_50_ = 3919 mg/kg, and, LD_50_ = 2500 mg/kg, respectively), may be or may not harmful if swallowed; one compound Dihydrokaempferol have drug toxicity class IV (LD_50_ = 2000 mg/kg) can be harmful if swallowed; and two compounds Germichrysone and Benzeneacetic acid have drug toxicity class III (LD_50_ = 221 mg/kg and LD_50_ = 300 mg/kg, respectively), can be toxic if swallowed (Table 4).

**Table 4:** Prediction of LD_50_ and toxicity class of compounds

Sr. No	Compounds	Predicted LD_50_	Predicted toxicity class
1	Germichrysone	221 mg/k	Class III
2	Benzeneacetic acid	300 mg/kg	Class III
3	Flavan-3-ol	2500 mg/kg	Class V
4	5,7,3',4'-Tetrahydroxy-6, 8-dimethoxyflavon	3919 mg/kg	Class V
5	Dihydrokaempferol	2000 mg/kg	Class IV
6	Epiafzelechin	2500 mg/kg	Class V

Class I (Fatal) if swallowed: (LD_50_ ≤ 5); Class II (fatal) if swallowed: (5 < LD_50_ ≤ 50); Class III (toxic) if swallowed (50 < LD_50_ ≤ 300); Class IV (harmful) if swallowed: (300 < LD_50_ ≤ 2000); Class V (may be harmful) if swallowed (2000 < LD_50_ ≤ 5000); Class VI (non-toxic) if swallowed (LD_50_ > 5000)

### Bioactivity score prediction of compounds

Bioactivity score prediction results showed that only one compound benzeneacetic acid was biologically inactive as enzyme, protease, kinase, GPCR, Ion channel modulator and nuclear receptor inhibitor. Among the other compounds examined, Germichrysone, Flavan-3-ol, and Dihydrokaempferol displayed moderate biological activity as kinase inhibitors.

**Fig. 1:**
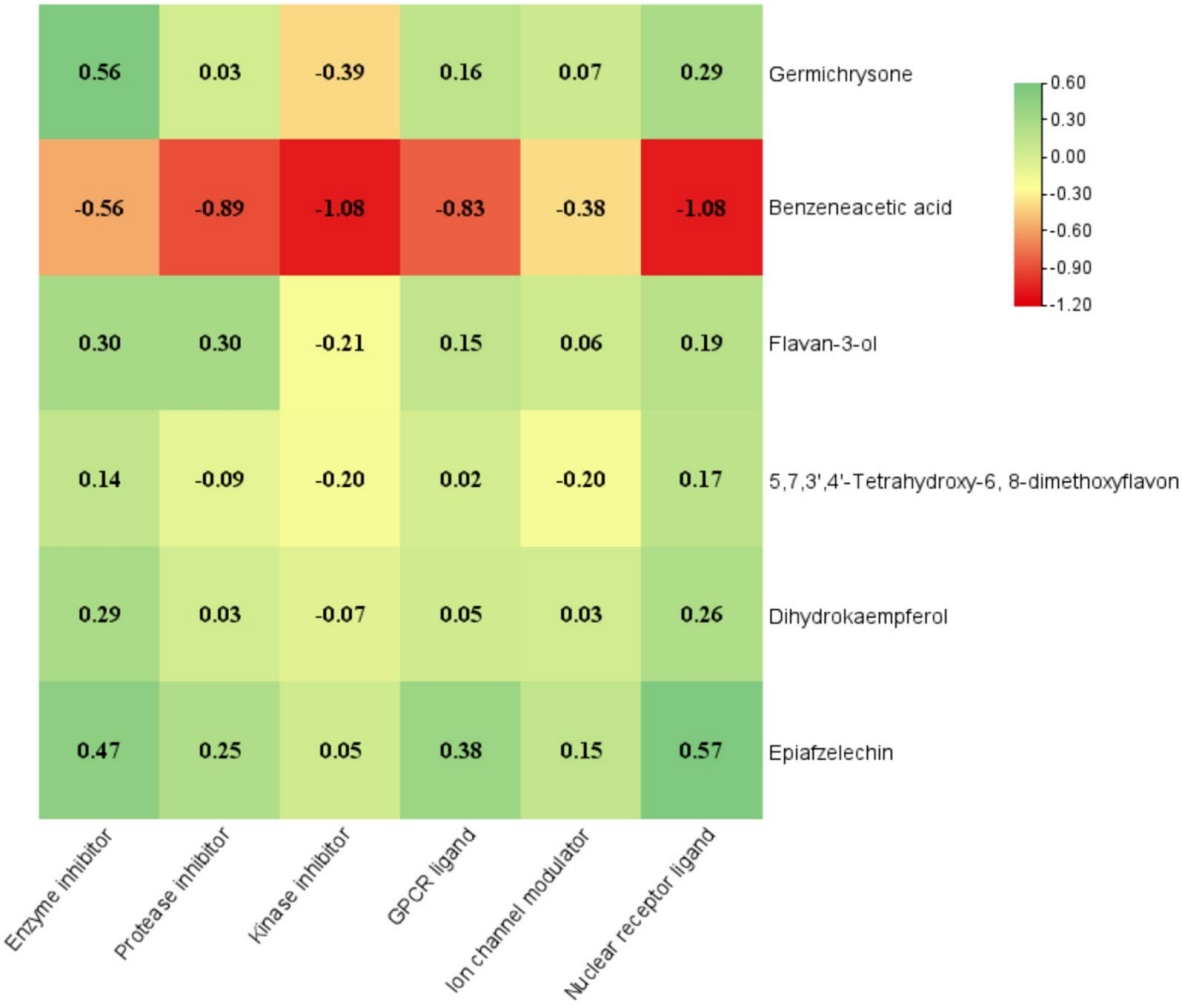
Bioactivity score prediction map of different compounds and activities receptor ligand; > 0.0 (active), −5.0 to 0.0 (moderately active) and below −5.0 (inactive)

On the other hand, 5,7,3',4'-Tetrahydroxy-6,8-dimethoxyflavon also exhibited moderate biological activity as proteases, kinases, and ion channel modulators inhibitor. The remaining compounds (Germichrysone, Flavan-3-ol, 5,7,3',4'-Tetrahydroxy-6,8-dimethoxyflavon, Dihydrokaempferol and Epiafzelechin) demonstrated high biological activity as enzyme inhibitors, protease inhibitors, GPCR ligands, ion channel modulators, kinase and nuclear receptor ligands (Fig. 1). Compound bioactivity refers to the ability of a chemical compound to produce a specific biological effects or response when interacting with a living organism or biological system (Hussein and Azeez 2023; Mukhtar and Khan 2023).

### Network pharmacology analysis

#### Identification of potential targets

**Fig. 2:**
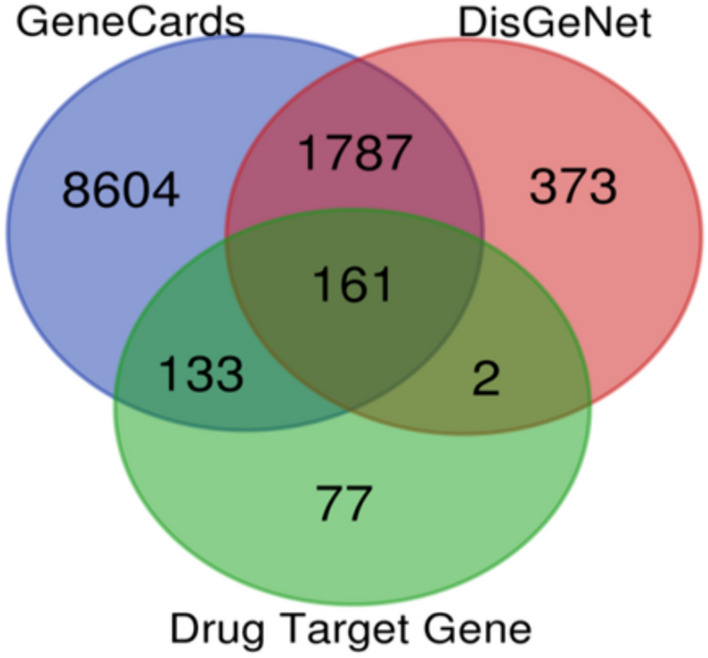
Venn diagram of targeted genes and drug target genes

Network pharmacology is an interdisciplinary approach that analyzes complex interactions between biological systems, drugs, and diseases to gain a holistic understanding of drug actions and discover novel therapeutic targets (Nogales et al. 2022; Yuan et al. 2022). The 662 targets were retrieved from 6 compounds through the SwissTargetPrediction. The potential targets of hypertension found in the databases GeneCard and DisGeNet were 10685 and 2323, respectively. Following the elimination of duplicates and the integration of hypertension-related targets, 161 common targets were identified, signifying potential intersections between compound targets and those associated with hypertension. These shared targets were regarded as potential targets for the selected plants in their hypertension-related actions (Fig. 2). A protein–protein interaction network reveals the intricate web of connections between various proteins in a cell, crucial for understanding cellular functions and disease mechanisms (Wang et al. 2022a, b, c; Rodina et al. 2023).

#### Construction of compound-target network

**Fig. 3:**
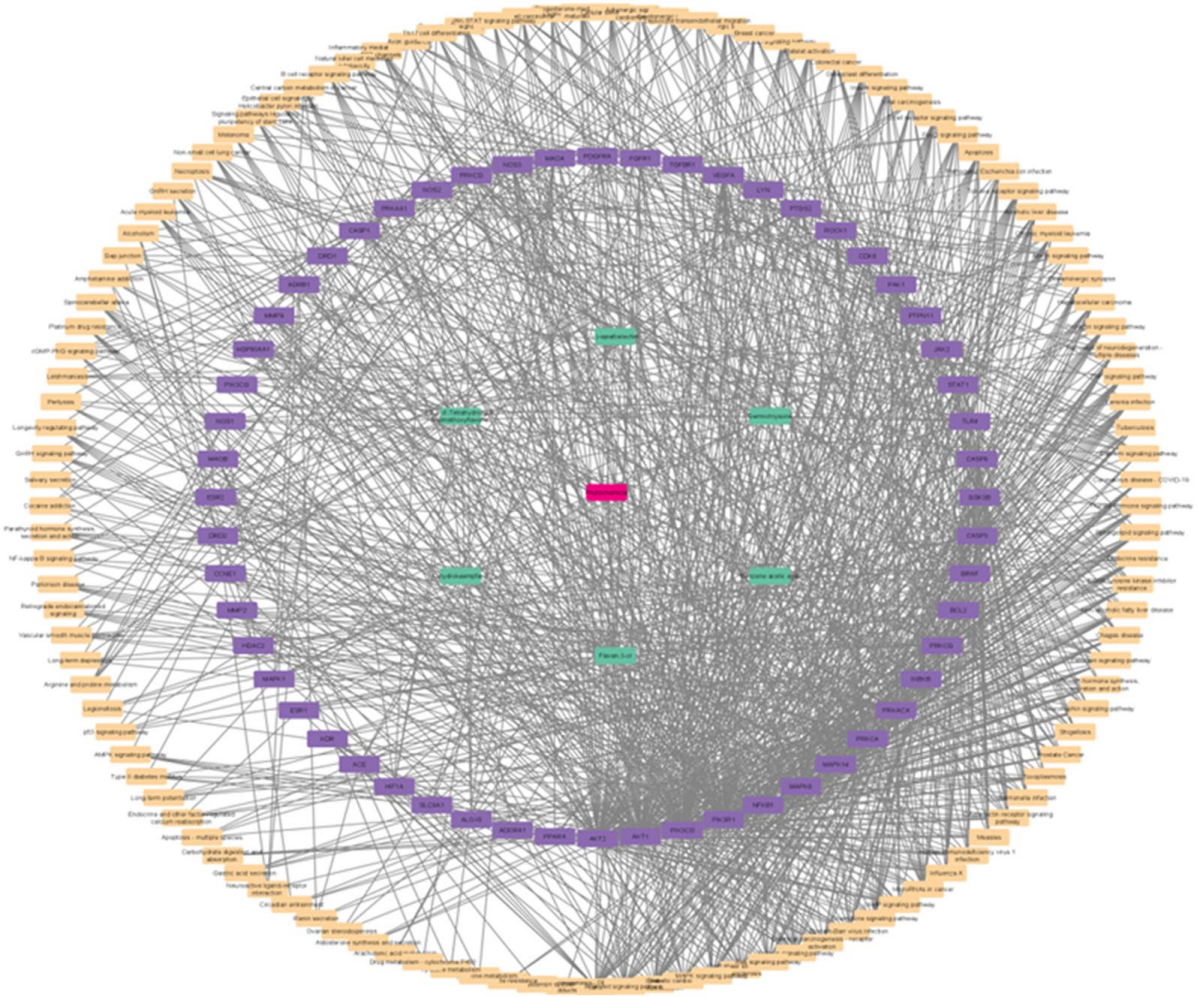
Compound-target Network

Compound-target network was constructed by using Cytoscape to analyze the interaction between the 6 active compounds and 161 potential targets (Fig. 3). In Fig. 3, the network green-colored nodes at the center represent the phytoconstituents; purple-colored nodes show the potential targets of hypertension and orange-colored nodes showing the targets function in different pathways regulation. Furthermore, the edges depict the interaction of chemicals and targets. Target-Compound network analysis shows that one active ingredient can affect many targets, while the same target may interact with more than one active compound. This reflects the multi-target and multi-components effects of the compounds in the medication for hypertension.

#### Construction of Protein–protein-Interaction (PPI) and hub genes

**Fig. 4:**
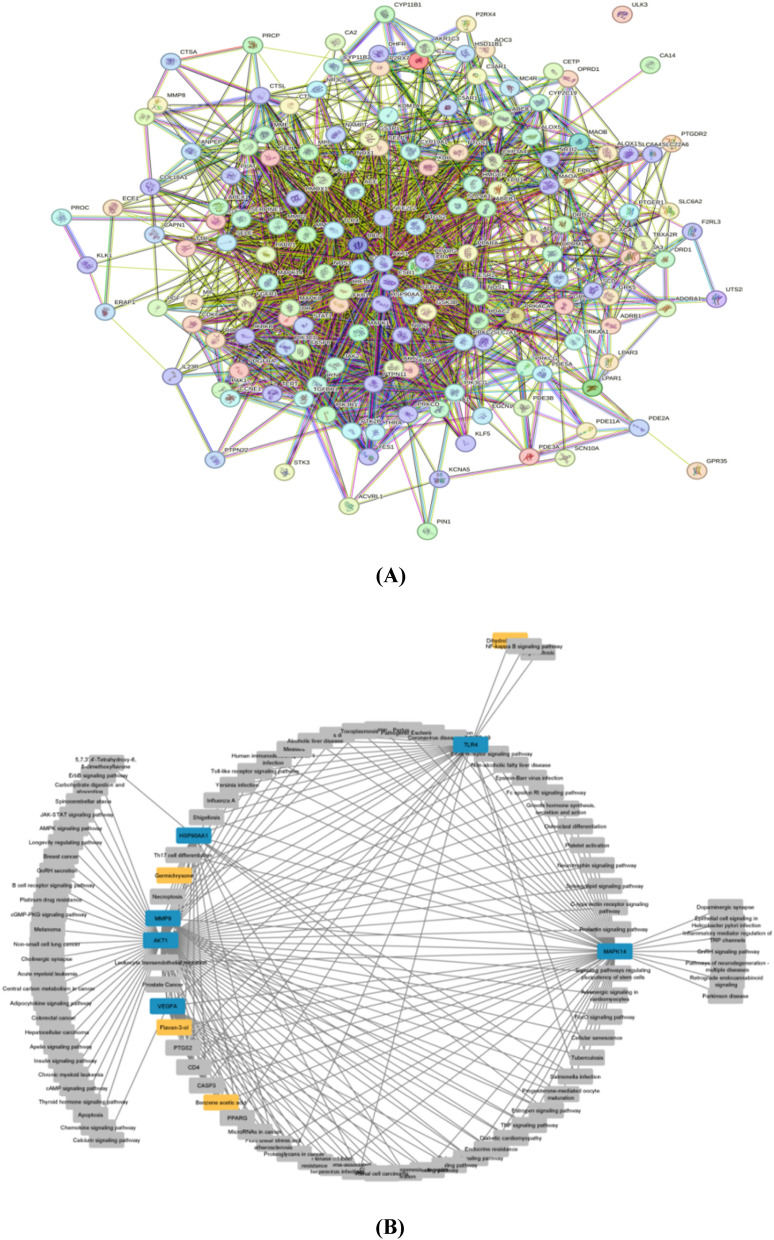
**A** Protein–protein Interaction Network **B** Compound-target-pathway Network

The Protein–protein-Interaction network of 161 common genes was constructed by using STRING database. After visualizing the PPI network in Cytoscape, 161 nodes with 1871 edges were found (Fig. 4A). The CytoHubba plugin was employed to identify hub genes, with an examination of 12 topological analysis methods for hub gene prediction. Among these 12 methods, the degree method was chosen to identify the top ranked hub genes. The following genes, namely AKT1, CASP3, HSP90AA1, MAPK14, MMP9, PPARG, PTGS2, TLR4, and VEGFA, emerged as the top-ranked genes due to their significantly high degree values (Table 5). After first stage node genes identification, four compounds Germichrysone, Flavan-3-ol, Benzeneacetic acid and Dihydrokaempferol was found effective against TLR4, MMP9, MAPK14, AKT1, VEGFA and HSP90AA1 (Fig. 4B.).

**Table 5:** Topological analysis of compounds and genes

Phytochemicals	Genes	Between-ness	Bottle-Neck	Closen-ess	CC	Degree	DMNC	Ec-Centricity	EPC	MCC	MNC	Radia-lity	Stress
5,7,3',4'-Tetrahydroxy-6, 8-dimethoxyflavo-ne	–	0	1	3.83333	0	1	0	0.16071	2.202	1	1	2.00893	0
–	AKT1	0	1	3.83333	0	1	0	0.16071	2.161	1	1	2.00893	0
Benzene acetic acid	–	0	1	3.83333	0	1	0	0.16071	2.258	1	1	2.00893	0
–	CASP3	0	1	3.83333	0	1	0	0.16071	2.2	1	1	2.00893	0
Dihydrokaemp-ferol	–	44	9	6.16667	0	5	0	0.21429	3.377	5	1	2.57143	44
Flavan-3-ol	–	30	9	5	0	2	0	0.32143	2.897	2	1	2.49107	30
Germichrysone	–	0	1	3.33333	0	1	0	0.16071	1.958	1	1	1.6875	0
–	HSP90AA1	0	1	3.33333	0	1	0	0.16071	1.934	1	1	1.6875	0
–	MAPK14	26	3	4.83333	0	3	0	0.21429	2.759	3	1	2.25	26
–	MMP9	0	1	2.08333	0	1	0	0.08929	1.695	1	1	1.33929	0
–	PPARG	6	2	2.83333	0	2	0	0.11905	2.095	2	1	1.60714	6
–	PTGS2	8	5	3	0	2	0	0.17857	2.222	2	1	1.69643	8
–	TLR4	6	2	2.83333	0	2	0	0.11905	2.074	2	1	1.60714	6
–	VEGFA	0	1	2.08333	0	1	0	0.08929	1.646	1	1	1.33929	0

*DMNC* Density of maximum neighborhood component, *MCC* maximal clique centrality, *EPC* edge percolated component, *MNC* Maximum neighborhood component, *CC* clustering coefficient

#### Construction of compound-target-pathway network

The mechanism of 4 compounds Germichrysone, Flavan-3-ol, Benzeneacetic acid and Dihydrokaempferol was studied in hypertension. For this purpose enriched pathways were selected by DAVID analysis and compound-target-pathway network was constructed with Cytoscape (Fig. 4B). The degree values determined the node color and size, while the active components' targets exhibited coordination through various paths, connecting with each other and contributing to the management of hypertension.

#### Gene ontology and pathway enrichment analysis

**Fig. 5:**
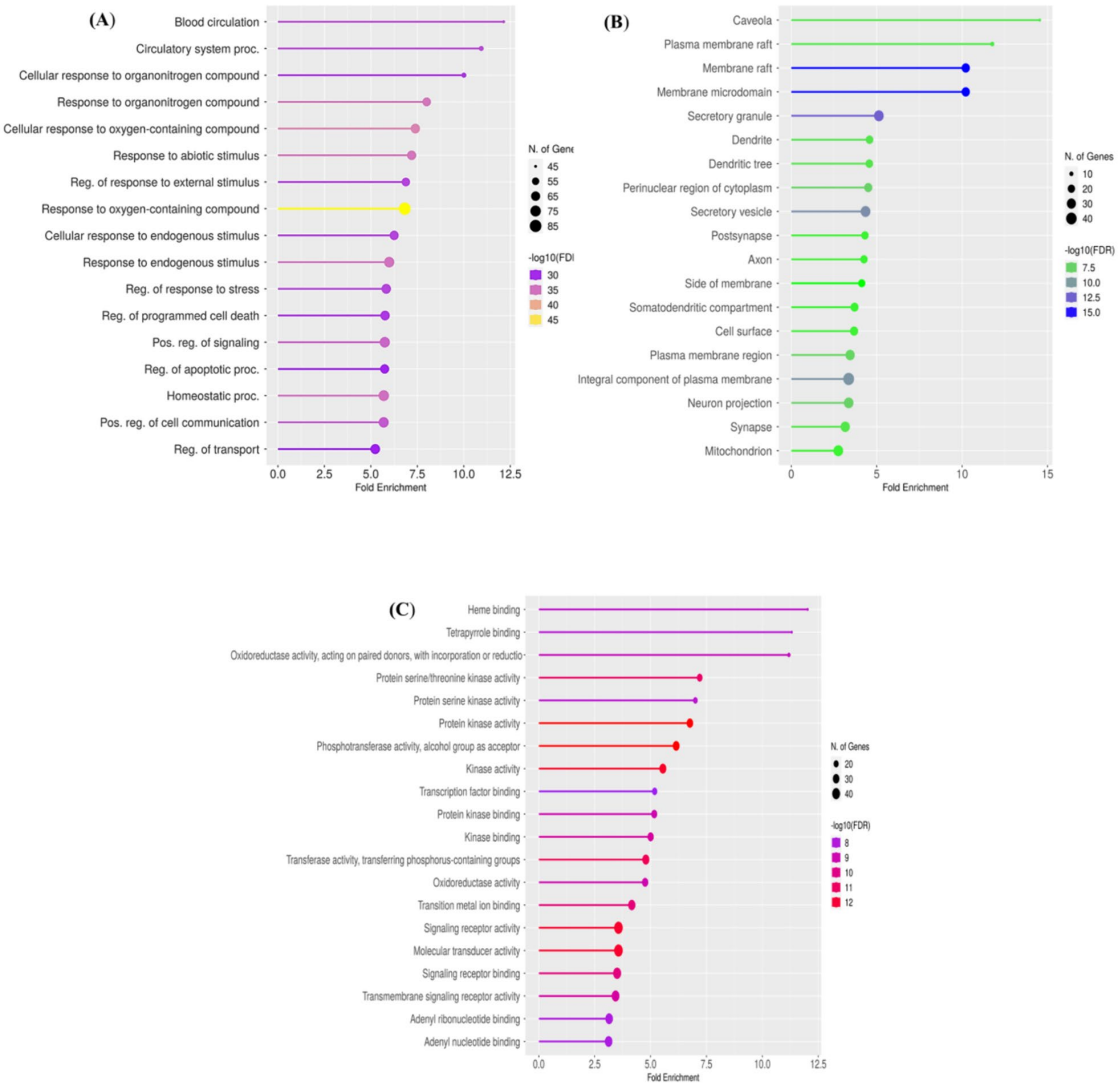
Gene ontology plots **A** Biological process **B** Cellular Components **C** Molecular function

Gene Ontology is a structured and standardized system used to categorize and describe the functions of genes and their products (Wang et al. 2022a, b, c). In order to clarify the molecular mechanisms through which active compounds enhance hypertension treatment, GO annotations and KEGG pathway analysis was conducted on a set of 161 targets associated with anti-hypertension activity. GO analysis recognized 386 biological processes (BP) (Fig. 5A), which include regulation of transport system, positive regulation of cell communication, positive regulation of signaling pathways, regulation of programmed cell death and blood circulation (Mabhida et al. 2021; Wei et al. 2022; Wang et al. 2023); 56 cellular components (CC) (Fig. 5B), which include integral component of plasma membrane, neuron projection, axon, cellular surface, secretory vesicle, synapse, membrane raft, perinuclear region of cytoplasm, dendrite and synapse (Ali et al. 2022; He et al. 2023; Wang et al. 2022a, b, c); and 110 molecular functions (MF) (Fig. 5C) such as protein serine/threonine kinase activity, which regulate vascular tone and renin–angiotensin–aldosterone system (RAAS), renal sodium handling and baroreceptors; phosphotransferase activity/alcohol group as acceptor; kinase activity; signaling receptor activity such baroreceptors, adrenergic receptors, endothelin receptors, Renin–angiotensin–aldosterone system receptors play crucial role in blood pressure regulation; oxidoreductase activity, which involves physiological processes that influence vascular function, endothelial health and cardiovascular homeostasis (Zeng et al 2017; Cui et al. 2018; Pradana et al. 2023). Applying the cutoff value p < 0.05 top 20 GO annotations (BP, CC, and MF) were selected to draw lollipop plots.

**Fig. 6:**
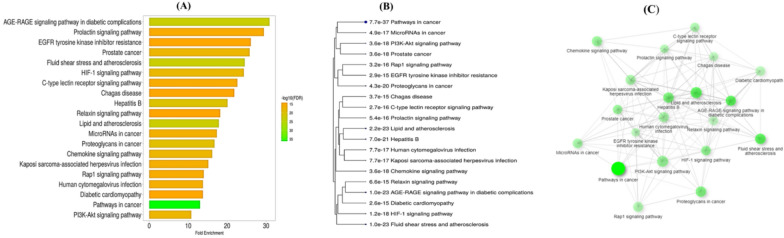
Enrichment analyses of targeted genes **A** KEEG pathways **B** clustering between pathways **C** pathways interaction network

KEEG analysis predicted 118 pathways regarding the hypertension, which include PI3K-Akt signaling pathway which regulate various cellular pathways in blood pressure regulation like endothelial function, inflammation and renal function; Rap1 signaling pathway which regulate vascular tone and endothelial cells to control blood pressure; EGFR tyrosine kinase inhibitor resistance like erlotinib or gefitinib increase blood pressure, inhibiting EGFR regulate blood vessels function; HIF-1 signaling pathway also referred to as Hypoxia-Inducible Factor 1 (HIF-1) signaling pathway, studies suggest that HIF-1 regulate Renin-angiotensin system and can affect the expression of renin; and AGE-RAGE signaling pathway in diabetic complications, chronic activation of the AGE-RAGE pathway contributes to endothelial dysfunction (Fig. 6A) (Di et al. 2018; Mabhida et al. 2021; He et al. 2023). A hierarchical clustering tree was constructed to summarize the correlation among significant pathways. Pathways with many shared genes are clustered together. In Fig. 6B bigger dots indicating more significant P-values. Furthermore, an interaction network was built between these enriched pathways to study the relationship. The network establishes connections between two pathways (nodes) if they possess 20% or more shared genes, with 20% being the default threshold. Nodes with a darker shade indicate more significantly enriched gene sets, while larger nodes signify larger gene sets. Thicker edges denote a higher degree of gene overlap (Fig. 6C). Applying the cutoff value p < 0.05 top 20 KEGG pathways were selected to draw bar plot, cluster tree and interaction network.

#### Gene/protein structure view by domain and motif analysis

**Fig. 7:**
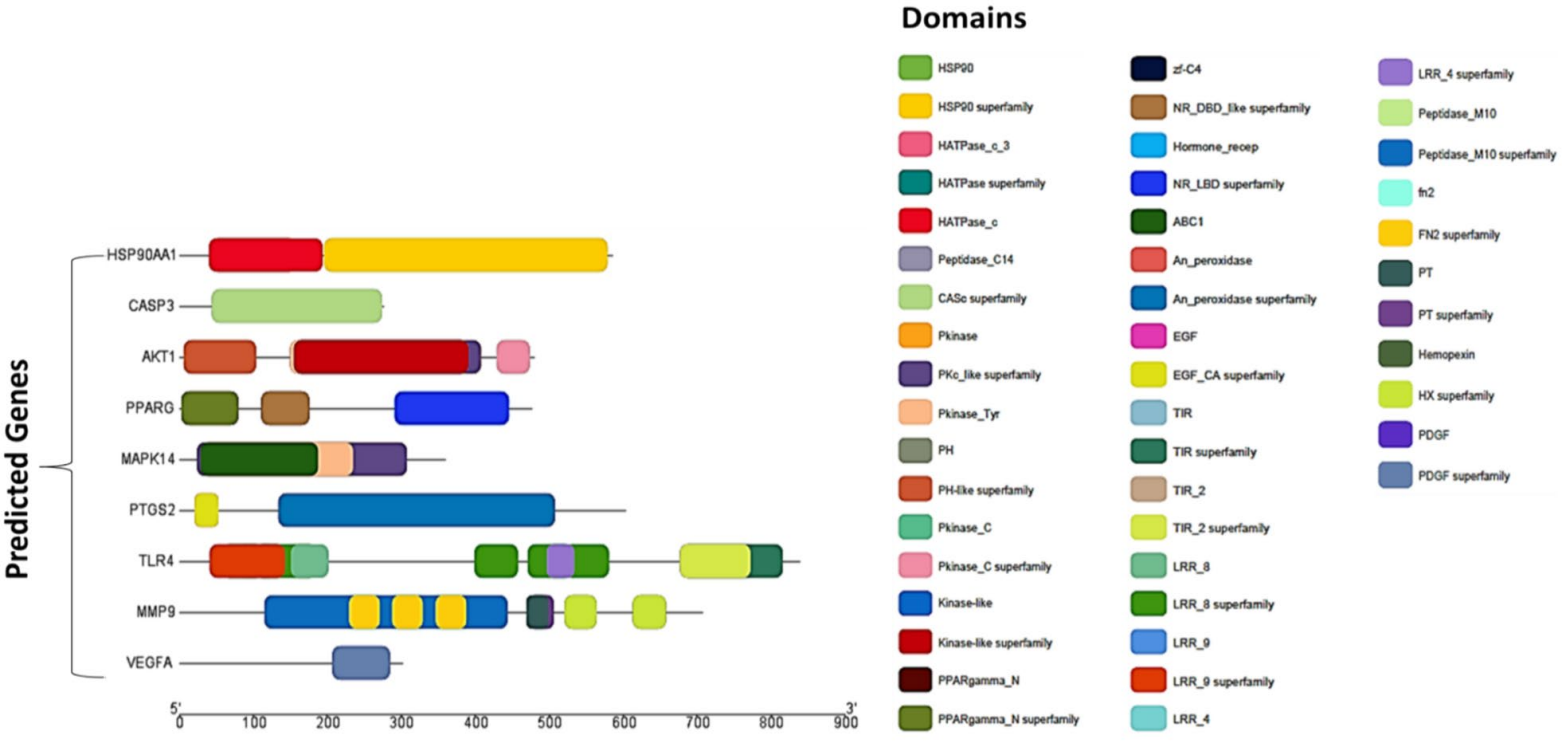
Conserved domains in top hub genes

Domain and motif analysis are techniques used to study the structure and function of gene/ proteins (Shen et al. 2020). Several genes may share common domains, domain superfamilies, and motifs, participating in various cellular, biological, and molecular functions (Jia et al. 2023). In the context of proteins, a domain is a distinct structural unit or region within the protein that has a specific function. Proteins are composed of one or more domains, each of which contributes to the overall function of the protein. Analysis revealed that different domains and domain super families were found involved in targeted genes cellular functions (HSP90, HSP90 Superfamily, HATPase_c_3, peptidase_C14, PKinase, PKc_like Superfamily, PKinase tyr, PH, PKinase_C_ Superfamily, PKinase_C, Kinase-like, Kinase_like superfamily, PPARgamma_N, Hormone_recep, NR_LBD superfamily, ABC1, An_peroxidase, TIR, TIR_2, LRR_4 superfamily, peptidase_M10, fn2 superfamily, PT, PDGF, PDGF superfamily), molecular functions (HSP90 Superfamily, HATPase_c_, HATPase_c_3, HATPase Superfamily, CASc superfamily, PKinase, PKc_like Superfamily, PH, PKinase_C_, Kinase-like, Kinase-like superfamily, PPARgamma_N, zf-C4, NR_DBD_like superfamily, Hormone_recep, NR_LBD superfamily, ABC1, An_peroxidase, An_peroxidase superfamily, TIR superfamily, TIR_2 superfamily, HX superfamily) and biological functions (HSP90, peptidase_C14, PKinase, PKc_like Superfamily, PKinase tyr, PH, PH-like superfamily, PKinase_C, PKinase_C Superfamily, PPARgamma_N_, PPARgamma_N_ Superfamily, NR_LBD superfamily, ABC1, An_peroxidase, An_peroxidase superfamily, EGF, EGF_CA superfamily, TIR, TIR_2, LRR_8, LRR_8 superfamily, LRR_9, LRR_9 superfamily, LRR_4 superfamily, Peptidase_M10, Peptidase_M10 superfamily, fn2, PT superfamily, Hemopexin, PDGF, PDGF superfamily) (Fig. 7).

HSP90, also known as Heat shock protein 90, highly conserved domain and protein family involved in cellular homeostasis, regulation of signal transduction and cancer biology (Birbo et al. 2021; López et al. 2021). HATPase_c_3, HATPase superfamily and HATPase_c play essential role in transcription, DNA unwinding and replication and protein degradation (Xue et al. 2023). Peptidase_ C14 is peptidase domain involved in catalyzing the hydrolysis of peptide bonds in proteins, protein degradation and regulation of cell signaling (Velilla et al. 2023). CASc or CRAL/TRIO and Sec14p superfamily is a group of structurally related proteins that play role in cellular processes such as lipid binding and transport, signal transduction, and cellular differentiation and development (Song et al. 2020). PKinase, PKc_ superfamily, PKinase, PKinase_C, and PKinase_tyr involve in both cellular and molecular functions such as signal transduction, cell cycle regulation, transcriptional regulation, RNA processing and stability (Ahuja et al. 2019; Roskoski 2020). Kinase_like/Kinase_like superfamily involve in protein–protein interaction, regulation of kinase activity, substrate recognition and cellular localization (Paul and Srinivasan 2020). PPARgamma_N superfamily play role in ligand binding, transcriptional activation and regulation of gene expression (Nakadai et al. 2023). Zf-C4/ zinger finger and fn2 domains involve in DNA binding and transcriptional regulation (Fisher et al. 2023). NR-DBD, LR-DBD superfamilies, ABC1 and HX domains play crucial role in cellular and molecular response such as nuclear receptors, transcriptional regulation and hormone sensing (Abdullah‐Zawawi et al. 2021). Hormone_recep and An_peroxidase domains are involve in molecular and biological functions like gene expression and oxidative stress (Molina et al. 2022). Peptidase_M10 and PDGF superfamilies domains are involve in cell signaling, immune response and extracellular matrix remodeling (Nageswara et al. 2019).

**Fig. 8:**
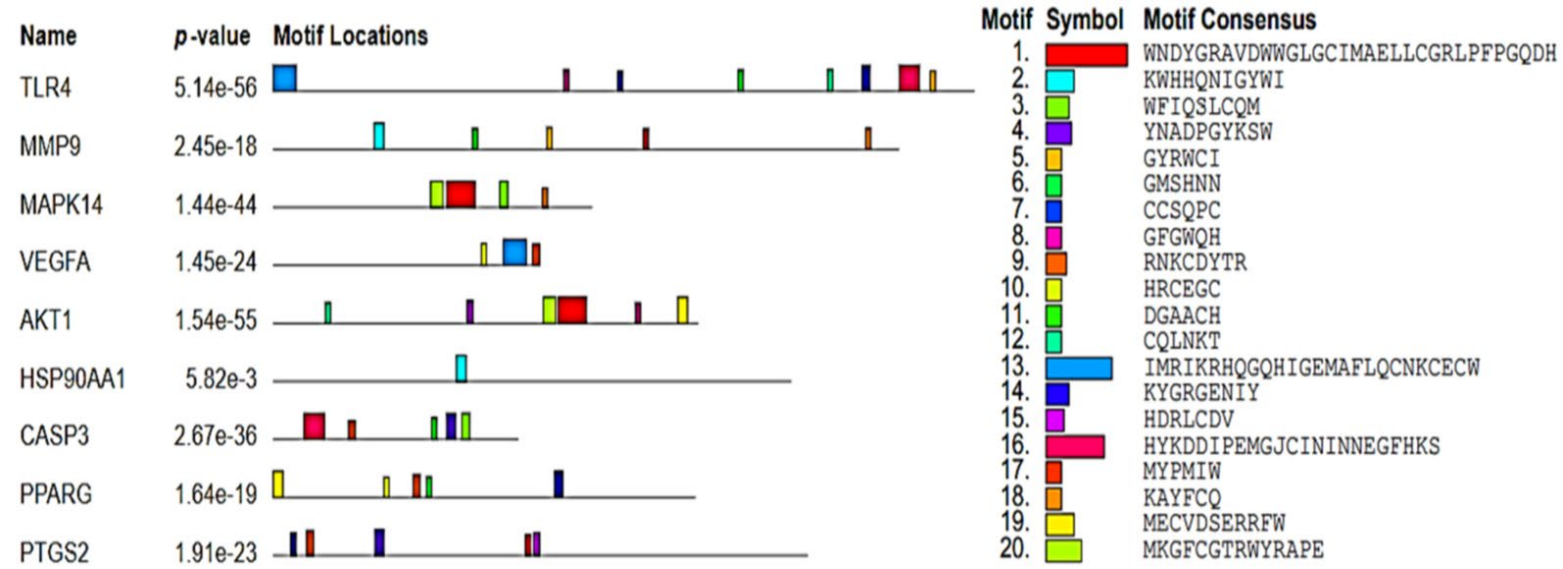
Conserved motifs in top hub genes

A motif is a short, conserved sequence pattern or structural element in nucleic acids or proteins, linked to specific functions or binding sites, and may exist as small regions within larger sequences, playing roles in functional activities like ligand or substrate binding in proteins In nucleic acids, motifs can be recognition sites for proteins. Figure 8 depicted top hub genes motif locations, symbol and motif consensus.

### Molecular docking

**Fig. 9:**
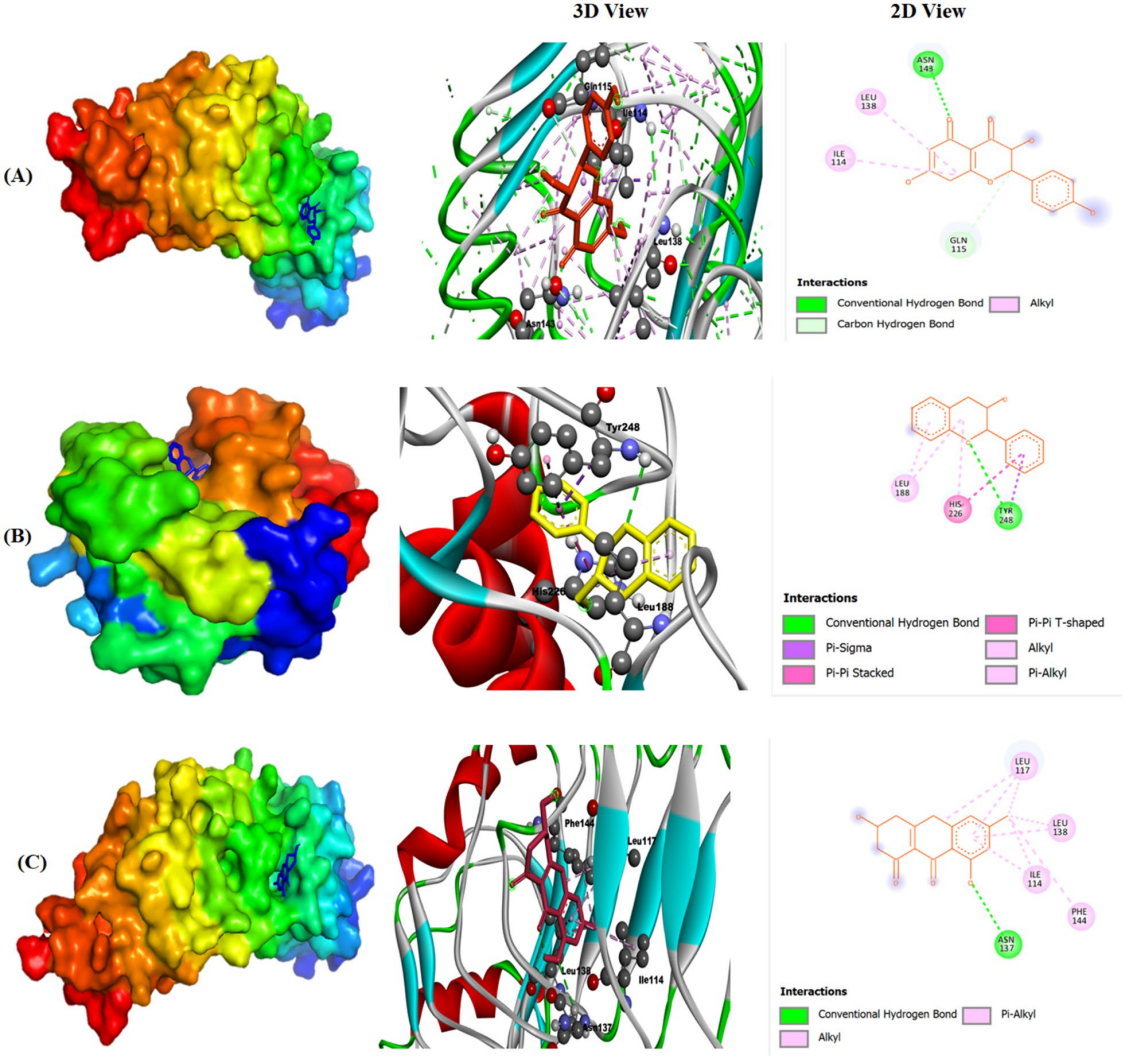
Molecular interactions and docking poses of selected phytoligands at different protein receptor sites **A** Dihydrokaempferol (TLR4) **B** Flavon-3-ol (MMP9) **C** Germichrysone (TLR4) representing docked protein structures and their Hu respective 3D and 2D model lines

Molecular docking is an essential component of drug discovery, as it anticipates the interactions between potential drug compounds and target proteins, thus facilitating the development of more precise and efficient medications (Nag et al. 2023). Molecular docking was employed to screen potential targets for components capable of reducing the occurrence of hypertension. Docking analysis successfully predicted a strong binding affinity between the components and the binding pockets of the target proteins. The four active components (Dihydrokaempferol, Flavan-3-ol, Benzeneacetic acid and Germichrysone) were docked with the six potential targets of hypertension (Table 6). The lower (more negative) the binding energy, the stronger the anticipated affinity for binding of the ligand against the target in molecular docking. The more negative the binding energy, the higher the expected affinity for ligand binding to the target during molecular docking (Sarkar et al. 2021; Kumar et al. 2023). A scoring function was employed to assess the positioning and order of docked structures, resulting in the generation of nine poses, from which the top one structure was chosen. The primary criterion for selecting the top structure was the number of hydrogen bonds present. Compounds with a higher count of hydrogen bonds were given preference. The phytochemicals showed binding energies for TLR4 ranging from − 5.9 kcal mol^−1^ to − 8.0 kcal mol^−1^. Gremichrysone showed the highest binding affinity with lowest binding energy (i.e., −8.0 kcal mol^−1^), followed by Dihydrokaepferol (−7.1 kcal mol^−1^). The highest number of interacting residues was observed in the interaction of TLR4 with both Germichrysone and Dihydrokampferol contain 4 interacting amino acid residues i.e. Leu 117, Leu 138, Ile 114, Phe 144 and Asn 137 and Asn 143, Leu 138, Ile 114 and Gln 115, respectively. The energies showed by phytochemical for MMP9 range from (−7.8 kcal mol^−1^ to −9.0 kcal mol^−1^), the binding affinity of Flavon-3-ol with MMP9 recorded was (−9.0 kcal mol^−1^), they have three amino acid residues i.e. Leu 188, His 226 and Tyr 248. The spectrum of energies exhibited by phytochemicals for MAPK14 varies from −4.2 kcal mol^−1^ to −7.8 kcal mol^−1^. The binding affinity of Flavon-3-ol for MAPK14 was noted -7.8 kcal mol^−1^, with highest number (i.e. nine) of interacting amino acid residues such as Ala157, Leu167, Thr106, Ala51, lys53, Ile84, Val38, His107 and Met109, followed by Benzene acetic acid (−5.6 kcal mol^−1^) have four interacting amino acid residues i.e. Ala51, Val38, Lys53 and Thr106. The energy showed by phytoligands for VEGFA range from −4.8 kcal mol^−1^ to −5.5 kcal mol^−1^. The binding affinity of Flavon-3-ol with VEGFA was note down -5.5 kcal mol^−1^ with two interacting amino acid residues i.e. Cys26 and Tyr25. The energy exhibited by phytoligands for AKT1 varies between −3.8 kcal mol^−1^ and −4.7 kcal mol^−1^. The Benzene acetic acid demonstrated a binding affinity of -4.7 kcal mol-1 with AKT1, with three specific amino acid residues involved: Ala50, Glu40, and Lys39. The energy showed by phytochemical for HSP90AA1 range from −5.6 kcal mol^−1^ to −6.7 kcal mol^−1^. The recorded binding affinity between Germichrysone and HSP90AA1 was −6.7 kcal mol^−1^, involving interaction with two specific amino acid residues: Asp93 and Asp54 (Table 6). The docked protein structures 2D and 3D model lines of three complexes Dihydro-TLR4, Flavon-MMP9 and Germich-TLR4 with greater binding affinity are given in Fig. 9 which was further validated by MD simulation. An examination of the interplay between protease and ligands revealed substantial influences from traditional hydrogen bonding, carbon hydrogen bonding, alkyl interactions, and pi–alkyl interactions (Mir et al. 2023a, b).

**Table 6:** The binding affinity of compounds and core targets

Sr. No	Compounds	Target	Target PDB ID	Target protein structure	Binding affinity (Kcal/mol)	Interacting amino acids residues
1	Dihydrokaempferol	TLR4	2Z62		−7.1	Asn 143, Leu 138, Ile 114 and Gln 115
2	Flavan-3-ol	MMP9	6ESM		−9.0	Leu 188, His 226 and Tyr 248
		MAPK14	6S9P		−7.8	Ala157, Leu167, Thr106, Ala51, Lys53, Ile84, Val38, His107 and Met109
		VEGFA	6ZBR		−5.5	Cys26 and Tyr 25
3	Benzene acetic acid	MAPK14	6S9P		−5.6	Ala51, Val38, Lys53 and Thr106
		AKT1	1UNQ		−4.7	Ala50, Glu40 and Lys39
4	Germichrysone	HSP90AA1	5J2X		−6.7	Asp93 and Asp54
		TLR4	2Z62		−8.0	Leu 117, Leu 138, Ile 114, Phe 144 and Asn 137

#### Molecular docking simulation

**Fig. 10:**
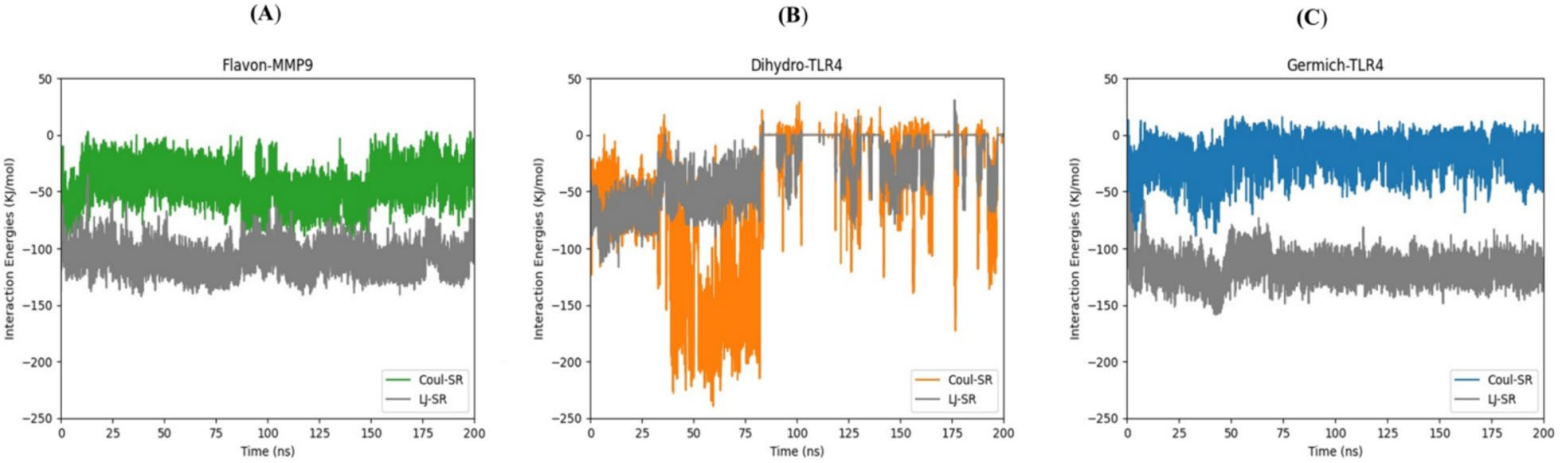
Time (ns) vs interaction energy (K/mol) plots of the molecular dynamic simulation of docking complexes involving the receptors **A** Flavon-MMP9 **B** Dihydro-TLR4 **C** Germi-TLR4

**Fig. 11:**
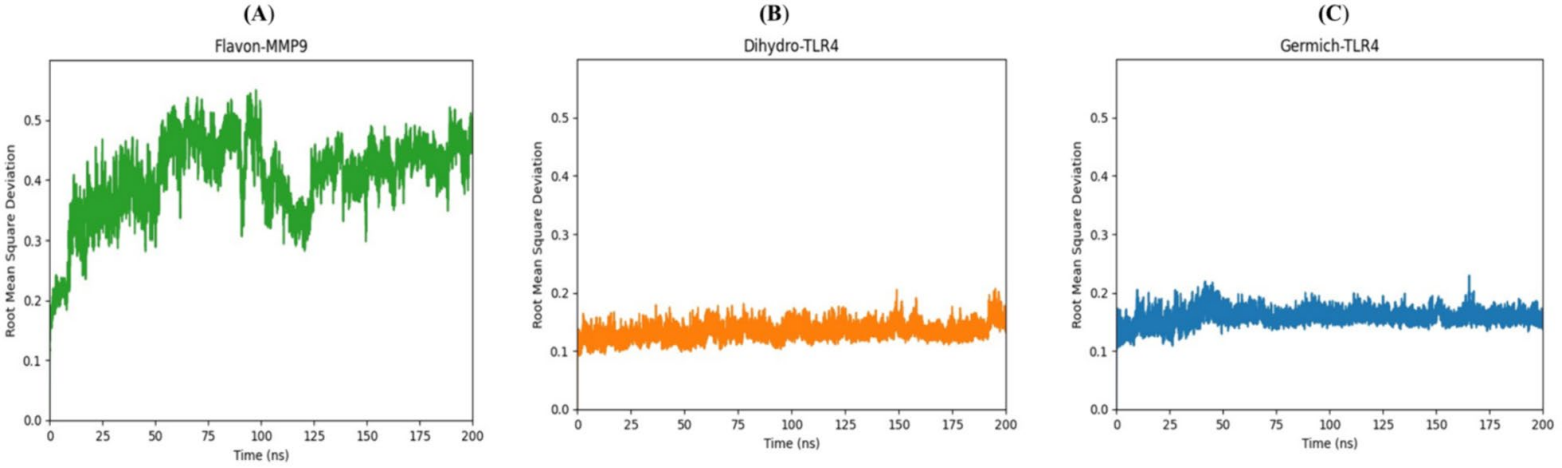
Time (ns) vs root mean square deviation plots of molecular dynamic simulation of docking complexes involving the receptors **A** Flavon-MMP9 **B** Dihydro-TLR4 **C** Germich-TLR4

**Fig. 12:**
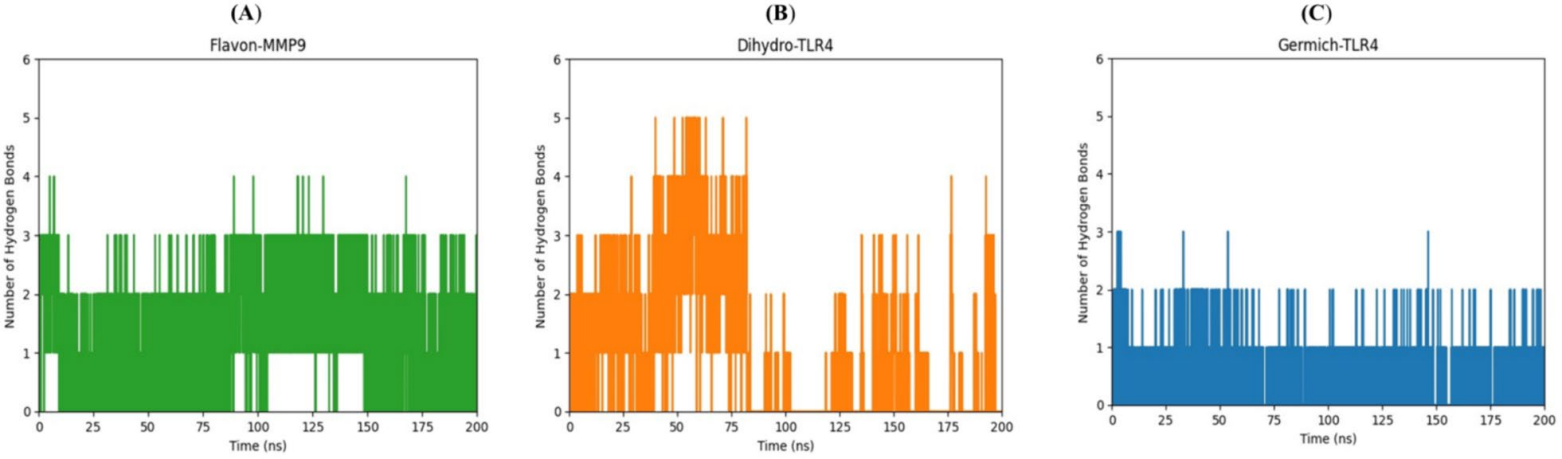
Time (ns) vs number of hydrogen bonds plots of molecular dynamic simulation of docking complexes involving the receptors **A** Flavon-MMP9 **B** Dihydro-TLR4 **C** Germich-TLR4

Molecular dynamics (MD) simulations are essential tools in the field of drug discovery and the evaluation of the structural stability of ligand–protein complexes (Fatullayev et al. 2023; Sayed et al. 2023). Through the simulation of the ever-changing behavior of these complexes, scientists can acquire valuable information regarding their binding stability and strength (Kumari et al. 2023; Sekaran et al. 2023). This information can be leveraged to fine-tune ligand structures, aiding in the assessment of potential drug effectiveness and the identification of the most favorable candidates for subsequent experimental trials (Patel et al. 2023). Based on the top-scoring results obtained from the docking complexes, we conducted molecular dynamics simulations for the following pairs: Flavan-3-ol and MMP9, dihydrokaempferol and TLR4, and Germichrysone and TLR4. Each complex underwent a 200 ns MD simulation to analyze the conformational dynamics of the core compounds and their respective targets. Various energy components were assessed, including potential energy, kinetic energy, average Coulombic short-range (Coul-SR) energy, and average Lennard–Jones short-range (LJ-SR) energy. Utilizing the final 200 ns of simulation trajectories, we calculated the interaction energy, which accounts for van der Waals and electrostatic interactions, to estimate their binding affinity at each site. Specifically, the Flavan-MMP9, Dihydro-TLR4, and Germich-TLR4 complexes exhibited average Coul-SR interaction energies of −43.8253, −49.0746, and −18.0598 kJ/mol, respectively. Moreover, the average LJ-SR interaction energies for these complexes were −110.202, −32.1595, and −119.129 kJ/mol, respectively. These findings indicate that the interaction between Flavan-MMP9 and Germich-TLR4 was stronger than that of the Dihydro-TLR4 complex, as depicted in Fig. 10A, B, and C. According to RMSD results, the Flavan-MMP9 complex began to rise from 0.2 nm at 25 ns and stabilized at around 100 ns, as shown in Fig. 11A. Dihydro-TLR4 remained stable at 0.1 nm with minor fluctuations and began to rise to 0.2 nm at 200 ns, as illustrated in Fig. 11B. The Germich-TLR4 complex reached 0.1 nm at 50 ns, as presented in Fig. 11C. Further, protein–ligand complexes are stabilized by hydrogen bonds (H-bonds). An analysis of H-bond interactions was conducted on the MD trajectories to determine the total number of H-bonds formed between protein–ligand complexes, shedding light on the binding affinity of ligands to proteins. The Flavan-MMP9, Dihydro-TLR4, and Germich-TLR4 complexes displayed H-bonds ranging from 0 to 4, 0 to 5, and 0 to 3, respectively. These outcomes revealed that, throughout the simulations, the total number of H-bonds in all protein–ligand complexes remained stable, as shown in Fig. 12A, B, and C.

#### Binding free energies of the complex by Molecular Mechanics Poisson-Boltzmann Surface Area (MM-PBSA)

**Fig. 13:**
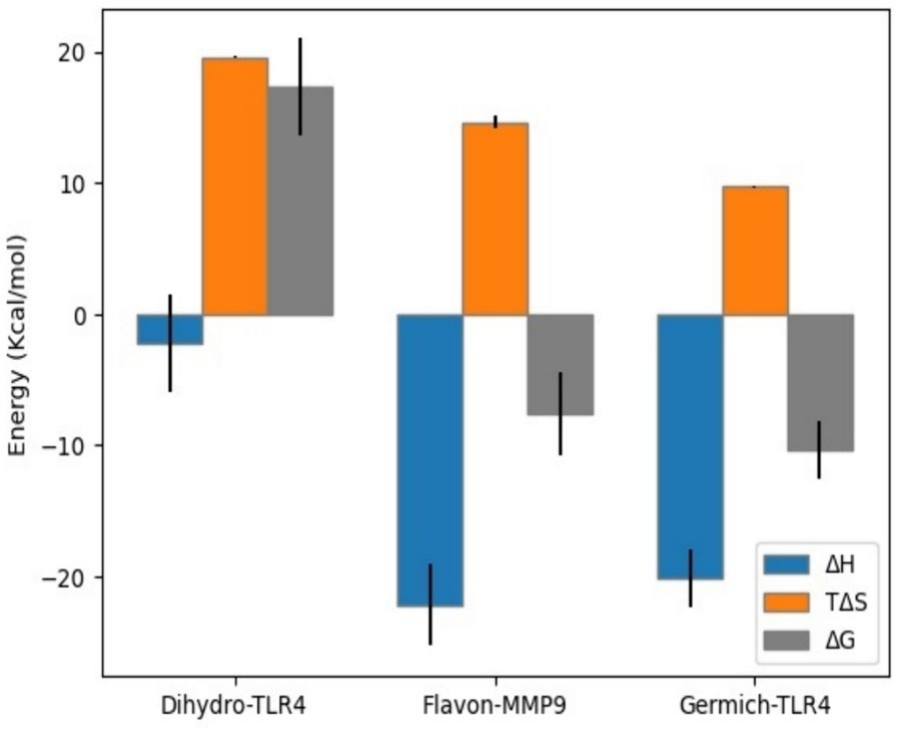
Binding free energies of ligand and protein complexes by MM-PBSA

Free binding energy calculations in molecular docking simulations were performed to estimate the strength of the interaction between a ligand and its target protein. The free binding energies (Δ*G*) calculated were 17.5, −4.5 and −9.7 kcal/mol in Dihydro-TLR4, Flavon-MMP9 and Germich-TLR4, respectively. These binding energies represent the thermodynamic stability of the ligand–protein complex; a more negative free binding energy indicates a more stable complex, suggesting a higher likelihood of the ligand binding tightly to the receptor (Fig. 8). In an isolated system, the total energy (ΔH) is conserved. ΔH calculated were 0.3, −20.4 and −19.5 kcal/mol in Dihydro-TLR4, Flavon-MMP9 and Germich-TLR4, respectively (Fig. 8). ΔH is essential in determining the equation of state (temperature, volume and pressure) for a system and understanding reaction mechanisms, reaction rates, and the stability of different chemical species. Moreover, the thermodynamic behavior of the system is measured by entropy TΔS. The entropic contribution to the binding free energy (TΔS) is significant factor; it helps in predicting and understanding the strength and specificity of molecular interactions. The values of TΔS calculated were 18.6, 4.8 and 7.7 kcal/mol in Dihydro-TLR4, Flavon-MMP9 and Germich-TLR4, respectively (Fig. 13).

Computational analytic technologies such as molecular docking and network pharmacology are essential for progressing clinical investigations because they offer priceless insights into the processes involved in drug discovery and development (Tao et al. 2020). Molecular docking facilitates the rational design of new therapies by predicting the binding affinity and manner of interaction between small compounds and target proteins. It streamlines the medication development process by enabling researchers to evaluate the safety and efficacy characteristics of possible therapeutic options (Kaur et al. 2019). Network pharmacology adds to this by explaining intricate relationships within biological systems, discovering synergistic effects, and identifying potential off-target effects or undesirable reactions (Li et al. 2023). Integrating these computational tools into clinical trials improves precision medicine initiatives by allowing for the identification of personalized treatment regimens matched to specific patient profiles, ultimately enhancing therapeutic outcomes and patient care (Collin et al. 2022).

Furthermore, network pharmacology and molecular docking have disadvantages, such as insufficient biological understanding, reliance on rigid models, and the requirement for experimental confirmation. The precision of scoring functions and the computational resource needs provide obstacles for molecular docking. Despite this, they continue to be useful tools in drug discovery when combined with experimental validation and other computational approaches (Kaushik et al. 2018).

## Conclusion

In this study, we delved into the potential mechanisms underlying the use of phytochemicals from three Fabaceae family species *Cassia fistula*, *Senna alexandrina* and *Cassia occidentalis* to treat hypertension. Our approach combined network pharmacology-based analysis with molecular docking and molecular dynamics (MD) simulation. Drug discovery analysis followed by network pharmacology analysis identified some important phytoconstituents germichrysone, benzeneacetic acid, Flavan-3-ol, 5,7,3',4'-Tetrahydroxy-6, 8-dimethoxyflavon, dihydrokaempferol, and epiafzelechin which revealed that these were the main constituents related to hypertension targets while TLR4, MMP9, MAPK14, AKT1, VEGFA and HSP90AA1 were the main hypertension-related molecular targets. 20 hypertension-related pathways were identified with the highest number of observed genes and lowest false discovery rate. Further, molecular docking studies showed that Dihydrokaempferol, Flavan-3-ol and Germichrysone possessed the highest binding energies towards all the targeted proteins (TLR4, MMP9). The study provided a comprehensive understanding of the suggested mechanism of action of compounds that may have potential use in hypertension treatment. The identified compounds Dihydrokaempferol, Flavan-3-ol belongs are active components of *Cassia fistula* and Germichrysone is the active components of *C. occidentalis*. In conclusion, our findings underscore the importance of exploring natural remedies as potential alternatives to conventional pharmacological interventions.

## Data Availability

The datasets utilized and examined in the present study can be obtained upon a reasonable request from the corresponding author.

## Abbreviations

ADMET: Absorption, distribution, metabolism, excretion, and toxicity
TLR4: Toll-like receptor 4
MMP9: Matrix metalloproteinase-9
VEGFA: Vascular endothelial growth factor A
AKT1: AKT Serine/threonine kinase 1
MAPK14: Mitogen-activated protein kinase
VEGFA: Vascular endothelial growth factor A
HSP90AA1: Heat shock protein 90 alpha family class A member 1
CYP1A2: Cytochrome P450 1A2
CYP2C19: Cytochrome P450 2C19
CYP2C9: Cytochrome P450 family 2 subfamily C member 9
CYP2D6: Cytochrome P450 family 2 subfamily D member 6
CYP3A4: Cytochrome P450 3A4, LD_50_: Lethal dose 50
STITCH: Search tool for interactions of chemicals
DAVID: The database for annotation, visualization. and integrated discovery
KEEG: Kyoto encyclopedia of genes and genomes
